# The Development and Validation of a Subscale for the School-Age Child Behavior CheckList to Screen for Autism Spectrum Disorder

**DOI:** 10.1007/s10803-022-05465-7

**Published:** 2022-02-15

**Authors:** Julia E. Offermans, Esther I. de Bruin, Aurelie M. C. Lange, Christel M. Middeldorp, Laura W. Wesseldijk, Dorret I. Boomsma, Gwen C. Dieleman, Susan M. Bögels, Francisca J. A. van Steensel

**Affiliations:** 1 UvA minds: Academic Treatment Center for Parents and Children, Banstraat 29, 1071 JW Amsterdam, the Netherlands; 2 Research Institute of Child Development and Education, University of Amsterdam, Nieuwe Achtergracht 127, 1018 WS Amsterdam, the Netherlands; 3 Viersprong: Institute for Studies on Personality Disorders, De Beeklaan 2, 4661 EP Halsteren, the Netherlands; 4 Child Health Research Centre, University of Queensland, 62 Graham Street, South Brisbane, QLD 4101 Australia; 5 Child and Youth Mental Health Service, Children’s Health Queensland Hospital and Health Service, 501 Stanley Street, South Brisbane, QLD 4101 Australia; 6 Department of Neuroscience, Karolinska Institutet Stockholm, Solnavägen 9, 171 65 Solna, Sweden; 7 Department of Psychiatry, Amsterdam Academic Medical Center—Location AMC—University of Amsterdam, Meibergdreef 9, 1105 AZ Amsterdam, the Netherlands; 8 Department of Biological Psychology, Free University of Amsterdam, Van der Boechorststraat 7, 1081 BT Amsterdam, the Netherlands; 9 Department of Child and Adolescent Psychiatry/Psychology, Erasmus University Medical Center-Sophia Children’s Hospital, Dr. Molewaterplein 40, 3015 GD Rotterdam, the Netherlands; 10 Department of Developmental Psychology, University of Amsterdam, Nieuwe Achtergracht 129-B, 1018 WS Amsterdam, the Netherlands

**Keywords:** Autism spectrum disorder (ASD), Screening, Child Behavior CheckList (CBCL), Achenbach System of empirically based Assessment (ASEBA), Children and adolescents

## Abstract

The first aim of this study was to construct/validate a subscale—with cut-offs considering gender/age differences—for the school-age Child Behavior CheckList (CBCL) to screen for Autism Spectrum Disorder (ASD) applying both data-driven (*N* = 1666) and clinician-expert (*N *= 15) approaches. Further, we compared these to previously established CBCL ASD profiles/subscales and DSM-oriented subscales. The second aim was to cross-validate results in two truly independent samples (*N* = 2445 and 886). Despite relatively low discriminative power of all subscales in the cross-validation samples, results indicated that the data-driven subscale had the best potential to screen for ASD and a similar screening potential as the DSM-oriented subscales. Given beneficial implications for pediatric/clinical practice, we encourage colleagues to continue the validation of this CBCL ASD subscale.

**Supplementary Information:**

The online version contains supplementary material available at 10.1007/s10803-022-05465-7.

Although ASD symptoms are already identifiable by 18 months of age (Chericoni et al., 2021), the diagnosis is often delayed until elementary school. For instance, in their study examining data of 2134 children included in two large family databases, Brett et al. (2016) found that the median age at ASD diagnosis in the UK was 4.5 years, which was comparable to the situation in the US. Delayed ASD diagnosis particularly pertains to children with ASD and cognitive functioning in the normal range, who are frequently diagnosed as late as during middle school. This is illustrated in the review of Daniels and Mandell (2014), who found that the median age at which children were diagnosed with Asperger’s disorder ranged from 7.4 to 11.2 years. Also, in many of these children, ASD may not be recognized at all. One of the studies supporting this claim was that of Kim et al. (2011), who screened for ASD in 55,266 South-Korean children, aged 7 to 12 years, in a sample consisting of both a high-probability group (drawn from a disability registry and special education schools) and a low-probability group (drawn from regular schools). They found an estimated ASD prevalence of 1.89% in the low probability group, as opposed to 0.75% in the high-probability group. This meant that two-thirds of the ASD cases came from the mainstream population—undiagnosed and untreated. Also, in their study examining case records of 2,867 children aged 6 to 12 years, who were registered at the Maccabi Child Development Center (MCDC) in Israel, Davidovitch et al. (2015) found that 221 children were diagnosed with ASD after the age of 6—even though their initial developmental evaluation (before the age of 6) came out negative for ASD. A delayed or missed ASD diagnosis has been associated with several factors, such as comorbid classifications (Supekar et al., 2017), the heterogeneity of ASD symptom composition, lower severity of ASD symptoms, lower levels of impairment (e.g., less language/communication deficits, less support needed by the child; less parental concern about initial symptoms; Daniels & Mandell, 2014), children’s ability to mask symptoms with learned strategies (APA, 2013), and the lack of adequate screening practices (Self et al., 2015). Also, detection as early as possible might prevent negative outcomes for both children (e.g., rejection by peers, harsh treatment by teachers, and inappropriate education) and their parents (e.g., frustration due to being told that there is nothing wrong with their child and not receiving appropriate support; Howlin & Asgharian, 1999). Therefore, routine-wise screening for ASD in the school-aged population is of great importance. In both community-based and clinical settings, the parent-rated school-age Child Behavior CheckList (CBCL 6-18; Achenbach & Rescorla, 2001) is an internationally used, reliable, and valid primary screening method for emotional, behavioral, and social problems in children aged 6 to 18 years (Rescorla et al., 2007). As it has been argued that this instrument contains several items that describe problem behaviors typical for children with ASD, several researchers have begun to investigate the ability of the school-age CBCL to identify childhood and adolescent ASD.

Initially, the capability of the school-age CBCL syndrome subscales—and specifically the Withdrawn/Depressed, Thought Problems, and Social Problems subscales—were examined to differentiate between children with and without ASD (e.g., Bölte et al. 1999; Duarte et al. 2003; Hoffmann et al., 2016). In two later studies, the discriminative power of certain combinations of syndrome subscales was investigated, which yielded the ASD profile (a combination of the Withdrawn/Depressed, Thought Problems, and Social Problems syndrome subscales; Biederman et al., 2010) and the WTP subscale (a combination of the Withdrawn/Depressed and Thought Problems syndrome subscales; Havdahl et al., 2016). Furthermore, two attempts have been made to develop a specific subscale, consisting of separate items to screen for ASD (Ooi et al., 2011; So et al., 2013). An overview of all ASD subscales derived from the school-age CBCL is presented in Table 1. Recently, Deckers et al. (2020) validated these ASD subscales in a sample of 132 children aged 6 to 18 years, of whom 75 were diagnosed with ASD (and cognitive functioning in the normal range) and 57 had another classification. The specific ASD subscales of Ooi et al. (2011) and So et al. (2013) were shown to have the best potential to distinguish between children with and without ASD.

**Table 1:** Items of the previously developed—i.e., the ASD profile, the WTP subscale, the specific ASD subscale by Ooi et al. (2011), and the specific ASD subscale by So et al. (2013)—and currently developed—i.e., the specific data-driven ASD subscale and clinician-expert ASD subscale—CBCL 6-18 subscales to screen for ASD

ASD profile (Biederman et al., 2010; *n *= 34) WTP subscale (Havdahl et al. 2016; *n* = 23)						
Withdrawn/Depressed syndrome subscale (*n* = 8)	Thought Problems syndrome subscale (*n* = 15)	Social Problems syndrome subscale (*n* = 11)	Specific ASD subscale by Ooi et al. (2011; *n* = 9)	Specific ASD subscale by So et al. (2013; *n *= 10)	Specific data-driven ASD subscale (*n* = 15)	Specific clinician-expert ASD subscale (*n *= 23)
There is little that he/she enjoys	Cannot get his/her mind off certain thoughts; obsessions	Clings to adults or too dependent	Acts too young for his/her age^d^	Acts too young for his/her age^d^	Acts too young for his/her age^d*^^	Acts too young for his/her age^d*^#^
Would rather be alone than with others	Deliberately harms self or attempts suicide	Complains of loneliness	Doesn’t get along with other kids^c^	Cannot get his/her mind off certain thoughts; obsessions^b^	There is little that he/she enjoys^a^	There is little that he/she enjoys^a#^
Refuses to talk	Hears sounds or voices that are not there	Doesn’t get along with other kids	Fears certain animals, situations, or places other than school^5^	Daydreams or gets lost in his/her thoughts^d^	Cannot get his/her mind off certain thoughts; obsessions^b^^	Bowel movements outside toilet^g^
Secretive, keeps things to self	Nervous movements or twitching	Easily jealous	Would rather be alone than with others^a^	Would rather be alone than with others^a^	Clings to adults or too dependent^c^	Cannot get his/her mind off certain thoughts; obsessions^b^#^
Too shy or timid	Picks nose, skin, or other parts of body	Feels others are out to get him/her	Nervous movements or twitching^e^	Poorly coordinated or clumsy^c^	Daydreams or gets lost in his/her thoughts^d^^	Daydreams or gets lost in his/her thoughts^d^#^
Underactive, slow moving, or lacks energy	Plays with own sex parts in public	Gets hurt a lot	Repeats certain acts over and over; compulsions^b^	Repeats certain acts over and over; compulsions^b^	Doesn’t get along with other kids^c,*^	Doesn’t eat well^g^
Unhappy, sad, or depressed	Plays with own sex parts too much	Gets teased a lot	Speech problem^c^	Speech problem^c^	Would rather be alone than with others^a*^^	Doesn’t get along with other kids^c*#^
Withdrawn, doesn’t get involved with others	Repeats certain acts over and over; compulsions	Not liked by other kids	Strange behavior^b^	Stares blankly^d^	Poorly coordinated or clumsy^c^^	Fears certain animals, situations, or places other than school^e*^
	Sees things that are not there	Poorly coordinated or clumsy	Withdrawn, doesn’t get involved with others^a^	Strange behavior^b^	Prefers being with younger kids^c^	Gets teased a lot^c^
	Sleeps less than most kids	Prefers being with younger kids		Withdrawn, doesn’t get involved with others^a^	Repeats certain acts over and over; compulsions^b*^^	Would rather be alone than with others^a*^#^
	Stores up too many things he/she does not need	Speech problem			Too shy or timid^a^	Not liked by other kids^c^
	Strange behavior				Stares blankly^d^^	Prefers being with younger kids^c#^
	Strange ideas				Strange behavior^b*^^	Repeats certain acts over and over; compulsions^b*^#^
	Talks or walks in sleep				Underactive, slow moving, or lacks energy^a^	Secretive, keeps things to self^a^
	Trouble sleeping				Withdrawn, doesn’t get involved with others^a*^^	Too shy or timid^a#^
						Stares blankly^d^#^
						Stores up too many things he/she does not need^b^
						Strange behavior^b*^#^
						Strange ideas^b^
						Stubborn, sullen, or irritable^f^
						Sudden changes in mood or feelings^f^
						Temper tantrums or hot temper^f^
						Withdrawn, doesn’t get involved with others^a*^#^

^a^Withdrawn/Depressed syndrome subscale

^b^Thought Problems syndrome subscale

^c^Social Problems syndrome subscale

^d^Attention Problems syndrome subscale

^e^Anxious/Depressed syndrome subscale

^f^Aggressive Behavior syndrome subscale

^g^Other Problems syndrome subscale

*Specific ASD subscale by Ooi et al. (2011)

^Specific ASD subscale by So et al. (2013)

#Specific data-driven ASD subscale

The specific ASD subscales of Ooi et al. (2011) and So et al. (2013) are comparable to the six DSM-oriented subscales (including Affective Problems, Anxiety Problems, Somatic Problems, Attention Deficit/Hyperactivity Problems, Oppositional Defiant Problems, and Conduct Problems) that have been developed by the Achenbach System of Empirically Based Assessment (ASEBA) for the school-age CBCL (Achenbach et al., 2001). These DSM-oriented subscales contain separate items that were rated by at least 14 out of 22 internationally recruited clinicians as being very consistent (opposed to not or somewhat consistent) with the concerning DSM IV-TR classification (e.g., Oppositional Defiant Disorder) or category (e.g., anxiety disorders). In a similar way, for the preschool-age version of the CBCL (CBCL 1.5-5), ASEBA has developed a DSM-oriented Autism Spectrum Problems subscale (Achenbach et al., 2000). However, such a DSM-oriented subscale for the school-age version of the CBCL by ASEBA is lacking. Also, the specific ASD subscales of Ooi et al. (2011) and So et al. (2013) have not been implemented in clinical practice. This might be due to limitations of the previous studies examining the ASD subscales for the school-age CBCL. First, in many studies, a small sample size (e.g., Biederman et al., 2010; Deckers et al., 2020; Havdahl et al., 2016) or a specific clinical control group (e.g., Ooi et al., 2011) was used, hampering generalizability of results. Second, in most studies, an additional sample to cross-validate results (i.e., to explore whether results generalize to an independent data set) was lacking (Biederman et al. 2010; Havdahl et al., 2016; Ooi et al., 2011) or the sample was split instead of including a truly independent cross-validation sample (So et al., 2013). Third, none of the studies differentiated between girls and boys or children and adolescents, nor did these include comparisons to the screening potential of ASEBA’s DSM-oriented subscales.

In the current study, we aimed to construct two specific ASD subscales for the school-age CBCL, the first following a data-driven approach [i.e., constructing a subscale based on data provided by parents, similar to the methods used by Ooi et al. (2011) and So et al. (2013)] and the second following a clinician-expert approach [i.e., constructing a subscale based on the opinions of clinicians, similar to the method used by ASEBA (Achenbach et al., 2001)]. Also, we aimed to validate the specific data-driven and clinician-expert ASD subscales and compare their screening potential to that of the syndrome subscales that formerly have been related to ASD, previously developed ASD subscales, and the DSM-oriented subscales. The second aim of this study was to cross-validate results in two truly independent samples.

## Method

### Participants

#### Sample 1

The first sample included a clinical (1A) and a typically developing (1B) subsample. The clinical subsample (1A) consisted of 1625 children aged 6 to 18 years (*M* = 10.86, *SD* = 2.93), 594 girls and 1031 boys. All children were referred to an academic mental health care center, located in Amsterdam, the Netherlands, for various emotional, behavioral, and/or social problems. Of these children, 270 had ASD and the others had different (*n* = 1290) or no (*n* = 65) DSM-IV-TR/5 classifications (see Table 2, also for comorbidity rates in the clinical subsample and in the ASD group specifically). Data was obtained from 1,573 mothers aged 24 to 63 years (*M* = 44.30, *SD* = 5.561) and 1,260 fathers aged 28 to 76 years (*M* = 46.74, *SD* = 6.07). For additional descriptives of the families included in Subsample 1A, see Table 3.

**Table 2:** Primary DSM-5 classifications, presence of classifications, and presence of comorbid classifications in subsample 1A (*n* = 1625)

	*n*	%
*Primary classification*		
Autism spectrum disorder	270	16.6
Attention deficit hyperactivity disorder	724	44.6
Anxiety disorders	203	12.5
Post-traumatic stress disorder	91	5.6
Affective disorders	61	3.8
Obsessive compulsive disorder and related disorders	56	3.4
Behavioral disorders	34	2.1
Disorder not otherwise specified	52	3.2
Other DSM-IV-TR/5 classification	69	4.2
No DSM-IV-TR/5 classification	65	4.0
*Presence of classifications*		
Autism spectrum disorder	270	16.6
Attention deficit hyperactivity disorder	773	47.6
Anxiety disorders	374	23.0
Post-traumatic stress disorder	142	8.7
Affective disorders	94	5.8
Obsessive compulsive disorder and related disorders	94	5.8
Behavioral disorders	60	3.7
Disorder not otherwise specified	58	3.6
Other diagnoses	251	15.4
*Presence of comorbid classifications*		
Complete clinical group	477	29.4
Autism spectrum disorder group	114	42.2

**Table 3:** Demographic statistics of subsample 1A (*n* = 1625)

			Mothers		Fathers	
	*N*	%	*N*	%	*N*	%
*Ethnic background*						
Caucasian	1438	88.5				
Surinam	44	2.7				
Moroccan and other Maghreb backgrounds	37	2.3				
Turkish and other middle Eastern backgrounds	29	1.8				
Indonesian and other Asian backgrounds	26	1.6				
Other	40	2.5				
Missing information	11	0.6				
*Living situation*						
With both parents	1064	65.5				
Co-parenting	199	12.2				
(Mainly) with mother	267	16.4				
(Mainly) with mother and new partner	49	3.0				
(Mainly) with father	12	0.7				
(Mainly) with father and new partner	8	0.5				
Missing information	26	1.7				
*Medication*						
Methylphenidate	84	5.2				
Melatonin	7	0.4				
Epilepsy-related medication	4	0.2				
Other	7	0.5				
Missing information	17	1				
None	1506	92.7				
IQ	938	57.7				
(*M*, *SD)*	*108.53*	*16.31*				
*Relation to the child*						
Biological parent			1513	96.2	1194	94.8
Foster parent			12	0.8	4	0.3
Adoptive parent			6	0.4	8	0.6
Stepparent			5	0.3	19	1.5
Other			5	0.3	3	0.2
Missing information			27	2	32	2.6
*Employment status*						
Working part-time			757	48.1	264	21.0
Working full-time			367	23.3	763	60.6
Stay-at-home parent			141	9.0	20	1.6
Unemployed/unable to work			97	6.2	47	3.7
Student			13	0.8	3	0.2
Other			165	10.5	123	9.8
Missing information			33	2.1	40	3.1
*Highest completed level of education*						
University			554	35.2	496	39.4
Applied university			526	33.4	318	25.2
Community college			174	11.1	127	10.1
High school			228	14.6	231	18.5
Elementary school			14	0.9	14	1.1
Other			46	2.9	38	3.0
Missing information			31	1.9	35	2.7

The typically developing subsample (1B) included 41 children aged 8 to 17 years (*M* = 12.85, *SD* = 2.49), 14 girls and 27 boys. Data was provided by 38 mothers aged 35 to 54 years (*M* = 44.32, *SD* = 4.28) and 34 fathers aged 37 to 59 years (*M* = 46.74, *SD* = 5.48). For further descriptives of Subsample 1B, see Telman et al. (2017).

#### Cross-validation samples

##### Sample 2

The second sample also contained two subsamples: A clinical (2A) and a typically developing (2B) subsample. The clinical subsample (2A) consisted of 2355 children aged 6 to 18 years (*M* =15.13, *SD* = 1.84), 912 girls and 1,443 boys. All children were referred to a mental health care organization with locations in various cities/towns in the Netherlands, mostly for behavioral problems. Of these children, 114 had ASD and the others had different (*n* = 2209; mainly behavioral disorders, namely *n* = 1579) or no (*n* = 32) DSM-IV-TR/5 classifications. In the complete clinical subsample, 3.7% of the children had at least one additional classification and in the ASD group particularly, comorbidity existed in 24.6% of the children. Data was collected from 2160 mothers and 1344 fathers. Data regarding parents’ age was unavailable.

The typically developing subsample (2B) included 90 children aged 7 to 18 years (*M* = 11.88, *SD* = 3.15), 43 girls and 47 boys. Data was obtained from 83 mothers aged 29 to 65 years (*M* = 42.37, *SD* = 5.57) and 45 fathers aged 34 to 56 years (*M* = 45.56, *SD* = 5.65). For further descriptives of Subsample 2B, see van Steensel et al. (2013).

##### Sample 3

The third sample also consisted of a clinical (3A) and a typically developing (3B) subsample. The clinical subsample (3A) comprised 857 children aged 6 to 18 years (*M* = 10.89, *SD* = 3.44), 368 girls and 489 boys. All children were referred to an academic mental health care center located in Amsterdam or Rotterdam, the Netherlands, for various emotional, behavioral, and/or social problems. Of these children, 220 had ASD and the others had different (*n* = 598) or no (*n* = 38) DSM-IV-TR/5 classifications. In the complete clinical subsample, 35.7% of the children had at least one co-morbid classification and in the ASD group specifically, this concerned 64.5% of the children. Data was provided by 667 mothers aged 22 to 61 years (*M* = 42.12, *SD* = 6.84) and 292 fathers aged 18 to 66 years (*M* = 45.31, *SD* = 7.01).

The typically developing subsample (3B) consisted of 29 children aged 8 to 18 years (*M* =12.24, *SD* = 2.66), 12 girls and 17 boys. Data was collected from 28 mothers aged 35 to 56 years (*M* = 43.93, *SD* = 4.76) and 21 fathers aged 38 to 54 years (*M* = 45.86, *SD* = 4.81). For further descriptives of Subsample 3B, see Telman, van Steensel, Maric, and Bögels (2018).

#### Clinicians Sample

The Clinicians Sample included 15 clinicians (all psychologists with at least a Master of Science degree, of which 8 also completed a postdoctoral training to be registered as a clinical psychologist)—employed at the mental health care center where the data of Subsample 1A were collected—aged 25 to 61 years (*M* = 32.93, *SD* = 9.14), 14 females and one male. On average, these employees had been working as clinicians for 9.14 years (*SD* = 8.62, range = 2 months–35 years). All clinicians worked with a varied clinical population (i.e., they provided diagnostic and treatment facilities to children with ASD and their families, but also to children with other classifications and their families).

### Instrument

The school-age version of the CBCL (CBCL 6-18; Achenbach & Rescorla, 2001; Verhulst & Van der Ende, 2001) is a standardized questionnaire to assess competencies as well as emotional, behavioral, and social problems. The part of the questionnaire concerning problems consists of 120 items, in which parents are asked to indicate on a three-point scale (0 = not true; 1 = somewhat/sometimes true; and 2 = very/often true) whether their child showed these symptoms during the past 6 months. The school-age CBCL consists of a Total Problems scale, two broad-band subscales (i.e., Internalizing Problems and Externalizing Problems), nine syndrome subscales (i.e., Withdrawn/Depressed, Thought Problems, Social Problems, Anxious/Depressed, Somatic Complaints, Attention Problems, Rule-Breaking Behavior, Aggressive Behavior, and Other Problems—the first three being of interest in the current study), and six DSM-oriented subscales (i.e., Affective Problems, Anxiety Problems, Attention Deficit/Hyperactive Problems, Oppositional Defiant Problems, Conduct Problems, and Somatic Problems—of which the latter was not validated in the current study). Sum scores were calculated, with higher scores reflecting more problems. Previous studies have established the psychometric properties of the school-age CBCL in both typically developing and clinical populations (Achenbach et al., 2008; de Groot et al., 1994; Ivanova et al., 2007), as well as in ASD samples (Hartini et al., 2016; Magyar & Pandolfi, 2017; Pandolfi et al., 2014). In all three samples of the current study, the internal consistency for the Total Problems scale was good (Cronbach’s *α* > 0.86). Reliability scores for the subscales can be found in Table 4. In addition, clinicians were asked—for each of the 120 school-age CBCL items—to indicate whether they felt the item did not (0) or did (1) characterize ASD.

**Table 4:** Descriptive statistics and results of the reliability analyses

	Sample 1 (*N* = 1666)			Sample 2 (*N* = 2445)			Sample 3 (*N* = 886)		
	*M (SD)*		*CA*	*M (SD)*		*CA*	*M (SD)*		*CA*
	DG	CG		DG	CG		DG	CG	
*Syndrome subscales*									
Withdrawn/depressed^a^	6.11 (3.38)	3.39 (2.84)	0.81	6.30 (2.77)	5.96 (2.99)	0.73	5.20 (3.26)	4.26 (3.34)	0.75
Thought problems^a^	6.41 (3.94)	4.47 (3.20)	0.73	7.77 (3.88)	5.73 (3.99)	0.78	6.42 (4.57)	4.93 (3.91)	0.69
Social problems^a^	6.40 (3.25)	4.14 (3.07)	0.75	6.67 (3.30)	5.51 (3.60)	0.79	6.95 (4.22)	4.73 (3.78)	0.77
*Previously developed ASD subscales*									
ASD profile by Biederman et al. (2010)^a^	18.93 (7.31)	12.00 (7.16)	0.86	20.89 (8.35)	17.18 (9.10)	0.89	18.26 (9.45)	13.54 (8.36)	0.84
WTP subscale by Havdahl et al. (2016)^a^	12.52 (5.54)	7.86 (5.05)	0.82	13.99 (6.03)	11.60 (6.20)	0.84	11.37 (6.34)	9.06 (5.91)	0.77
Specific ASD subscale by Ooi et al. (2011)^a^	5.80 (2.70)	3.03 (2.34)	0.68	5.10 (2.51)	3.77 (2.52)	0.65	6.30 (3.72)	3.82 (2.92)	0.66
Specific ASD subscale by So et al. (2013)^a^	7.37 (3.11)	4.33 (2.64)	0.71	6.02 (2.73)	4.90 (3.05)	0.71	7.47 (4.42)	4.92 (3.29)	0.71
*DSM-oriented subscales*									
Affective problems^b^	10.03 (4.68)	4.72 (3.43)	0.78	10.05 (4.73)	7.28 (4.25)	0.79	7.28 (4.84)	5.69 (4.15)	0.74
Anxiety problems^c^	5.29 (2.52)	2.86 (2.23)	0.78	5.08 (2.90)	3.02 (2.27)	0.71	5.48 (3.21)	3.64 (2.67)	0.72
Attention/hyperactive problems^d^	8.05 (2.65)	4.42 (2.91)	0.84	7.47 (2.49)	5.68 (2.98)	0.76	7.94 (3.55)	5.78 (3.75)	0.84
Oppositional defiant problems^e^	6.72 (1.90)	3.40 (2.36)	0.82	5.17 (2.48)	4.45 (2.59)	0.81	4.88 (2.29)	3.38 (2.44)	0.69
Conduct problems^f^	NA	NA	0.81	9.98 (5.86)	9.25 (5.35)	0.85	NA	NA	0.82
*Currently developed ASD subscales*									
Specific data-driven ASD subscale^a^	11.51 (4.41)	6.62 (3.97)	0.80	9.59 (4.09)	7.89 (4.51)	0.79	10.85 (5.83)	7.71 (4.90)	0.79
Specific clinician-expert ASD subscale^a^	16.31 (5.80)	10.22 (5.75)	0.84	15.52 (6.11)	12.85 (6.59)	0.86	16.83 (7.72)	11.78 (6.81)	0.83

*CA* Cronbach’s Alpha; *DG* disorder group (including all children with the DSM-IV-TR/5 classification of interest); *CG* control group (including all children with other or no DSM-IV-TR/5 classifications); *ASD profile* a combination of the Withdrawn/Depressed, Thought Problems, and Social Problems syndrome subscales; *WTP subscale* a combination of the Withdrawn/Depressed and Thought Problems syndrome subscales; *Specific ASD subscale* an ASD subscale consisting of separate CBCL 6-18 items; *NA* not available, due to there being no children with Conduct Disorder included in the concerning sample

^a^DG = children with a DSM-classification of Autism spectrum disorder

^b^DG = children with a DSM-classification of any affective disorder (e.g., Major depressive disorder or Persistent depressive disorder)

^c^DG = children with a DSM-classification of any anxiety disorder (e.g., Generalized anxiety disorder, Social anxiety disorder, or Specific phobia)

^d^DG = children with a DSM-classification of Attention deficit hyperactivity disorder, including all sub-types

^e^DG = children with a DSM-classification of Oppositional defiant disorder or any related behavioral disorder (excluding Conduct disorder)

^f^DG = children with a DSM-classification of Conduct disorder

### Procedure

Children’s DSM-IV-TR/5 classifications were determined by multidisciplinary teams consisting of licensed psychologists and psychiatrists, who used information from multiple sources (i.e., parents, teachers, and children) and assessments (i.e., interviews, observations, questionnaires, and psychiatric/neuropsychological evaluations). When classifications had previously been established (i.e., when children were only referred for treatment), those were used in the current study. This pertained particularly to Sample 2. For the mental health care center where the data of Subsamples 1 and 2A was collected, an inclusion criterion for referral was that children’s level of cognitive functioning was at a minimum of below average (i.e., IQ > 70), as estimated based on their school performances.

The current study was approved by the ethical committee of the University of Amsterdam. Parents, as well as children aged 12 years or older, gave active (Sample 1, Subsample 2B, and Sample 3) or passive (Subsample 2A) informed consent. Parents completed online questionnaires at home, providing information on their child’s problem behaviors (for the clinical subsamples: Before the intake session at the mental health care center took place).

### Analyses

#### Development of the specific ASD subscales

The first aim of this study was to develop an ASD subscale out of separate items of the school-age CBCL. We used two approaches: (1) a data-driven one in which we examined school-age CBCL data provided by parents, and (2) a clinician-expert one in which we examined opinions of clinicians.

##### Specific data-driven ASD subscale

For the development of the specific data-driven ASD subscale, in the first sample, we tested which of the school-age CBCL items best differentiated between children with and without ASD, by using multilevel models consisting of repeated measurements of informants (i.e., mothers and fathers; level one-units), which were nested in children (level two-units). Advantages of multilevel analysis are that nested data is accounted for and missing data is handled, so that imputation is not necessary (Kreft & de Leeuw, 1998). Therefore, the multilevel analyses were based on all available data, including the cases for which only one informant had completed the school-age CBCL. In 120 separate multilevel models, the categorical variable ‘primary classification’ was added as a predictor to test all school-age CBCL items. This way, we could identify on which of these items the ASD group scored significantly higher compared to each of the 10 comparison groups (i.e., nine clinical groups—based on their primary classification—and one typically developing group; see Table 2 for the categories of primary classifications in clinical Subsample 1A). Children’s age and gender were included as covariates, as previous studies have demonstrated that both variables affect CBCL scores (e.g., Achenbach & Rescorla, 2001; Hartley & Sikora, 2009; Ooi et al., 2011). We chose an inclusion criterion of 70%, meaning that an item was included in the specific data-driven ASD subscale when the ASD group scored significantly higher on that item than at least 7 of the 10 comparison groups. Because of the large size of the first sample (as well as of both cross-validation samples) and the fact that we conducted multiple analyses, we chose an *α* level of < 0.001. Although we did not perform a priori power analyses, post-hoc power analyses confirmed that we had sufficient power to conduct the multilevel analyses (as well as to conduct the ROC-analyses).

##### Specific Clinician-Expert ASD Subscale

For each school-age CBCL item, we counted how many clinicians indicated that item as characteristic for ASD. We chose a similar inclusion criterion as in the data-driven approach, meaning that an item was included in the specific clinician-expert ASD subscale when at least 11 out of the 15 clinicians (73.33%) indicated that item as typical for ASD.

#### Validation of the specific ASD subscales

The second aim of this study was to validate the specific data-driven and clinician-expert ASD subscales (i.e., to test whether these subscales correctly classified children with and without ASD). To do so, we used raw school-age CBCL cores, as recommended by Achenbach and Rescorla (2001). Also, when data was provided by both mother and father, average scores were used. First, we calculated the Cronbach’s *α.* Values between 0.50 and 0.59 can be interpreted as poor, between 0.60 and 0.69 as questionable, between 0.70 and 0.79 as acceptable, between 0.80 and 0.89 as good, and between 0.90 and 1.0 as excellent (Cohen, 1988). Second, we calculated the Area Under the Curve (AUC), which is the area under the Receiver Operating Characteristic (ROC) curve. The ROC curve plots sensitivity (i.e., the percentage of correct positive classifications) against specificity (i.e., the percentage of correct negative classifications) for all possible cut-off scores. The AUC can range from 0.50 (chance level) to 1.0 (perfect fit). Values between 0.50 and 0.69 can be interpreted as poor, between 0.70 and 0.79 as fair, between 0.80 and 0.89 as good, and between 0.90 and 1.0 as excellent (Ferdinand, 2008). As the AUCs of the syndrome and ASD subscales were correlated (i.e., in all cases, the classification and control group consisted of children with and without ASD, respectively), DeLong et al. test (1988) was applied to compare discriminative power of the specific data-driven and clinician-expert ASD subscales to that of the syndrome and previously developed ASD subscales. Contrary, the make-up of the classification and control group differed by DSM-oriented subscale (e.g., the Anxiety Problems subscale distinguished between children with and without anxiety disorders, whereas the Attention Deficit/Hyperactive Problems subscale distinguished between children with and without ADHD). Therefore, comparisons of different ROC curves (Hanley & McNeil, 1982) were conducted when AUC scores for the specific data-driven and clinician-expert ASD subscales were compared to those for the Affective Problems, Anxiety Problems, Attention Deficit/Hyperactive Problems, Oppositional Defiant Problems, and Conduct Problems subscales. ROC analyses were used to establish the optimal cut-off scores. Additionally, we calculated sensitivity and specificity related to these cut-off scores. Following ASEBA, we developed cut-off scores for girls and boys separately, as well as for children and adolescents separately. Additionally, we developed both subclinical and clinical cut-off scores. As this study concerned the development of screening instruments, we based the subclinical cut-off scores on high sensitivity (approximately 80%) and the clinical cut-off scores on an optimal equilibrium between sensitivity and specificity, for all gender and age groups separately (Appendix: Table A).

To determine which subscale discriminated best between children with and without ASD, we repeated the analyses [i.e., ROC-analyses and DeLong et al.’s test (1988)] for the syndrome and previously developed ASD subscales. Additionally, to determine whether the specific data-driven and clinician-expert ASD subscales performed similarly to the DSM-oriented subscales, we also repeated the analyses (i.e., ROC-analyses and comparisons of different ROC-curves) for the Affective Problems, Anxiety Problems, Attention Deficit/Hyperactive Problems, Oppositional Defiant Problems, and Conduct Problems subscales. To determine sensitivity and specificity for the syndrome subscales, previously developed ASD subscales, and DSM-oriented subscales, we applied the cut-off scores established by ASEBA or the cut-off scores determined in previous studies (Appendix: Tables B, C, and D).

## Results

### Development and evaluation of the specific ASD subscales in the first sample

#### Development of the specific ASD subscales

##### Specific data-driven ASD subscale

Multilevel analyses yielded 15 school-age CBCL items on which the ASD group scored significantly higher than at least 7 of the 10 comparison groups. Of the items included in this specific data-driven ASD subscale, which are presented in Table 1, five were from the Withdrawn/Depressed, four were from the Social Problems, three were from the Thought Problems, and three were from the Attention Problems syndrome subscale. Between the specific data-driven ASD subscale and the specific ASD subscales developed by Ooi et al. (2011; n = 9 items) and So et al. (2013; n = 10 items), six and nine items overlapped, respectively.

##### Specific clinician-expert ASD subscale

Table 1 also shows the 23 items that at least 11 of the 15 clinicians indicated as characteristic for ASD. Of the items included, five were from the Withdrawn/Depressed, five were from the Thought Problems, three were from the Social Problems, three were from the Attention Problems, three were from the Aggressive Behavior, two were from the Other Problems, and one was from the Anxious/Depressed syndrome subscale. Between the specific data-driven and clinician-expert ASD subscales, 12 items overlapped. Besides, between the specific clinician-expert ASD subscale and the specific ASD subscales of Ooi et al. (2011) and So et al. (2013), seven and eight items overlapped, respectively. The specific data-driven and clinician-expert ASD subscales also overlapped by classifying children in the same way (i.e., as having ASD or not) for over 80% (Appendix: Table E).

#### Reliability and ROC Analyses

The internal consistency of both the data-driven and clinician-expert ASD subscale was good (Table 4). ROC analyses indicated that both the data-driven and clinician-expert ASD subscale could significantly discriminate between children with and without ASD: AUC scores could be interpreted as good and fair for the specific data-driven and clinician-expert ASD subscale, respectively.

#### Comparison to the syndrome and previously developed ASD subscales

The internal consistency of both the specific data-driven and clinician-expert ASD subscale was higher than that of the Thought Problems and Social Problems syndrome subscales, as well as the specific ASD subscales of Ooi et al. (2011) and So et al. (2013). Also, it was comparable to that of the Withdrawn/Depressed syndrome subscale and the combinations of syndrome subscales (i.e., the ASD profile of Biederman et al. [2010] and the WTP subscale of Havdahl et al. [2016]; Table 4). DeLong et al.’s (1988) test for pairwise comparisons of ROC curves confirmed that the discriminative power of both the data-driven and clinician-expert ASD subscale was higher compared to that of the (combinations of) syndrome subscales, but similar to that of the specific ASD subscale of Ooi et al. (2011). Also, the specific data-driven ASD subscale had a higher ability of discriminating between children with and without ASD compared to the specific ASD subscale of So et al. (2013), but this was not the case for the specific clinician-expert ASD subscale (Tables 8 and 9; Appendix: Tables F and G). When using the subclinical cut-off scores (Appendix: Tables A and B), sensitivity values were higher but specificity values were lower for both the specific data-driven and clinician-expert ASD subscale compared to those for the syndrome subscales, as well as those for the ASD profile of Biederman et al. (2010; Table 5). When using the clinical cut-off scores (Appendix: Table A), sensitivity and specificity values for both the specific data-driven and clinician-expert ASD subscale were similar to those for the WTP subscale of Havdahl et al. (2016), the specific ASD subscale of Ooi et al. (2011), and the specific ASD subscale of So et al. (2013; Table 5).

**Table 5:** Results of the ROC analyses conducted in Sample 1 (*N *= 1666)

	AUC	CI	*p*	Sensitivity	Specificity
*Syndrome subscales*					
Withdrawn/depressed	0.74	0.71–0.77	< 0.001		
Subclinical				66.7	71.3
Clinical				42.2	87.0
Thought problems	0.65	0.62–0.69	< 0.001		
Subclinical				55.6	63.5
Clinical				38.9	79.8
Social problems	0.70	0.67–0.74	< 0.001		
Subclinical				45.9	79.7
Clinical				20.0	92.0
*Previously developed ASD subscales*					
ASD profile by Biederman et al. (2010)	0.76	0.73–0.79	< 0.001	56.3	78.5
WTP subscale by Havdahl et al. (2016)	0.74	0.71–0.77	< 0.001	76.3	58.4
Specific ASD subscale by Ooi et al. (2011)	0.79	0.76–0.82	< 0.001	75.9	68.5
Specific ASD subscale by So et al. (2013)	0.77	0.74–0.80	< 0.001	79.3	59.6
*DSM-oriented subscales*					
Affective problems	0.82	0.78–0.86	< 0.001		
Subclinical				81.9	60.8
Clinical				63.8	77.9
Anxiety problems	0.77	0.74–0.80	< 0.001		
Subclinical				63.3	73.4
Clinical				44.5	86.4
Attention deficit/hyperactive problems	0.82	0.80–0.84	< 0.001		
Subclinical				49.3	87.0
Clinical				21.6	96.1
Oppositional defiant problems	0.86	0.81–90	< 0.001		
Subclinical				73.3	82.8
Clinical				46.7	91.3
Conduct problems	NA	NA	NA		
Subclinical				NA	NA
Clinical				NA	NA
*Currently developed ASD subscales*					
Specific data-driven ASD subscale	0.80	0.77–0.82	< 0.001		
Subclinical				85.2	54.7
Clinical				74.8	67.9
Specific clinician-expert ASD subscale	0.78	0.75–0.81	< 0.001		
Subclinical				83.7	58.5
Clinical				76.7	65.9

*AUC* Area under the Curve; *CI* AUC 95% confidence interval; *sensitivity* e.g., the specific data-driven ASD subscale was able to correctly classify 85.2% (subclinical cut-off score) or 74.8% (clinical cut-off score) of children with ASD as having an ASD classification; *specificity* e.g., the specific data-driven ASD subscale was able to correctly classify 54.7% (subclinical cut-off score) or 67.9% (clinical cut-off score) of children without ASD as having no ASD classification; *ASD profile* a combination of the Withdrawn/Depressed, Thought Problems, and Social Problems syndrome subscales; *WTP subscale* a combination of the Withdrawn/Depressed and Thought Problems syndrome subscales; *Specific ASD subscale* an ASD subscale consisting of separate CBCL 6-18 items; *NA* not available, due to there being no children with Conduct Disorder included in this sample

#### Comparison to the DSM-oriented subscales

The internal consistency of both the specific data-driven and clinician-expert ASD subscale was comparable to that of the DSM-oriented subscales (Table 4). Generally, the comparisons of different ROC curves demonstrated that both the specific data-driven and clinician-expert ASD subscale had similar levels of discriminative power as the DSM-oriented subscales. However, there were two exceptions: (1) the Oppositional Defiant Problems subscale had a higher discriminative ability than both the specific data-driven and clinician-expert ASD subscale and (2) the Attention Deficit/Hyperactive Problems subscale had a higher discriminative ability than the specific clinician-expert ASD subscale (Tables 5, 8 and 9; Appendix: Tables F and G). When comparing performance rates, we found that for both the specific data-driven and clinician-expert ASD subscale, sensitivity scores were higher but specificity scores were lower than those for the DSM-oriented subscales (Table 5).

### Validation of the specific ASD subscales in the cross-validation samples

#### Comparison to the syndrome and previously developed ASD subscales

In both cross-validation samples, the internal consistency of both the specific data-driven and clinician-expert ASD subscale was good, higher compared to that of the specific ASD subscales of Ooi et al. (2011) and So et al. (2013), and comparable to that of the (combinations of) syndrome subscales (Table 4). AUC scores for both the specific data-driven and clinical-expert ASD subscale reflected poor ability to discriminate between children with and without ASD in both cross-validation samples (Tables 6 and 7). However, this also pertained to the syndrome and previously developed ASD subscales [with an exception for the specific ASD subscale of Ooi et al. (2011) in Sample 3]. Generally, DeLong et al.’s (1988) tests for pairwise comparisons of ROC curves revealed that the ability of both the specific data-driven and clinician-expert ASD subscale to discriminate between children with and without ASD was higher or comparable to that of the syndrome and previously developed ASD subscales (Tables 8 and 9; Appendix: Tables F and G). In both cross-validation samples, comparable sensitivity and specificity rates were found for the specific data-driven ASD subscale, the specific clinician-expert ASD subscale, the syndrome subscales, and the previously developed ASD subscales (Tables 6 and 7).

**Table 6:** Results of the ROC analyses conducted in Sample 2 (N = 2445)

	AUC	CI	*p*	Sensitivity	Specificity
*Syndrome subscales*					
Withdrawn/depressed	0.53	0.48–0.58	0.263		
Subclinical				60.4	46.1
Clinical				30.2	69.7
Thought problems	0.65	0.60–0.70	< 0.001		
Subclinical				79.2	44.0
Clinical				50.0	66.9
Social problems	0.60	0.55–0.65	< 0.001		
Subclinical				58.7	54.2
Clinical				30.8	80.0
*Previously developed ASD subscales*					
ASD profile by Biederman et al. (2010)	0.62	0.57–0.68	< 0.001	65.2	51.6
WTP subscale by Havdahl et al. (2016)	0.61	0.56–0.66	< 0.001	81.4	34.8
Specific ASD subscale by Ooi et al. (2011)	0.66	0.61–0.71	< 0.001	67.6	53.4
Specific ASD subscale by So et al. (2013)	0.62	0.57–0.67	< 0.001	70.5	51.2
*DSM-oriented subscales*					
Affective problems	0.67	0.59–0.74	< 0.001		
Subclinical				83.3	36.8
Clinical				68.8	57.3
Anxiety problems	0.71	0.63–0.80	< 0.001		
Subclinical				62.2	69.9
Clinical				45.9	81.5
Attention deficit/hyperactive problems	0.67	0.65–0.70	< 0.001		
Subclinical				52.1	68.1
Clinical				16.0	89.9
Oppositional defiant problems	0.58	0.55–0.60	< 0.001		
Subclinical				40.8	66.3
Clinical				19.1	87.2
Conduct problems	0.53	0.47–0.60	0.284		
Subclinical				70.9	33.8
Clinical				37.2	68.7
*Currently developed ASD subscales*					
Specific data-driven ASD subscale	0.62	0.57–0.67	< 0.001		
Subclinical				72.3	45.2
Clinical				57.4	57.1
Specific clinician-expert ASD subscale		0.56–0.68	< 0.001		
Subclinical				75.6	38.7
Clinical				71.1	46.8

*AUC* Area under the Curve; *AUC CI* 95% confidence interval; *sensitivity* e.g., the specific data-driven ASD subscale was able to correctly classify 72.3% (subclinical cut-off score) or 57.4% (clinical cut-off score) of children with ASD as having an ASD classification; *specificity* e.g., the specific data-driven ASD subscale was able to correctly classify 45.2% (subclinical cut-off score) or 57.1% (clinical cut-off score) of children without ASD as having no ASD classification; *ASD profile* a combination of the Withdrawn/Depressed, Thought Problems, and Social Problems syndrome subscales; *WTP subscale* a combination of the Withdrawn/Depressed and Thought Problems syndrome subscales; *Specific ASD subscale* an ASD subscale consisting of separate CBCL 6-18 items

**Table 7:** Results of the ROC analyses conducted in Sample 3 (*N* = 886)

	AUC	CI	*p*	Sensitivity	Specificity
*Syndrome subscales*					
Withdrawn/depressed	0.59	0.55–0.64	< 0.001		
Subclinical				59.9	59.9
Clinical				39.2	77.9
Thought Problems	0.60	0.55–0.64	< 0.001		
Subclinical				55.4	56.1
Clinical				46.2	72.6
Social problems	0.66	0.62–0.70	< 0.001		
Subclinical				49.0	69.4
Clinical				30.1	86.2
*Previously developed ASD subscales*					
ASD profile by Biederman et al. (2010)	0.65	0.60–0.70	< 0.001	52.0	68.3
WTP subscale by Havdahl et al. (2016)	0.61	0.56–0.66	< 0.001	69.6	49.9
Specific ASD subscale by Ooi et al. (2011)	0.70	0.66–0.74	< 0.001	72.9	53.2
Specific ASD subscale by So et al. (2013)	0.67	0.63–0.72	< 0.001	71.1	52.7
*DSM-oriented subscales*					
Affective problems	0.60	0.53–0.67	0.007		
Subclinical				63.9	47.1
Clinical				44.4	66.6
Anxiety problems	0.67	0.62–0.72	< 0.001		
Subclinical				65.4	58.6
Clinical				48.4	73.5
Attention deficit/hyperactive problems	0.66	0.62–0.70	< 0.001		
Subclinical				51.7	66.2
Clinical				30.2	83.4
Oppositional defiant problems	0.68	0.62–0.74	< 0.001		
Subclinical				33.7	81.5
Clinical				27.5	92.2
Conduct problems	NA	NA	NA		
Subclinical				NA	NA
Clinical				NA	NA
*Currently developed ASD subscales*					
Specific data-driven ASD subscale	0.66	0.61–0.70	< 0.001		
Subclinical				73.9	46.9
Clinical				68.6	55.6
Specific clinician-expert ASD subscale	0.69	0.64–0.75	< 0.001		
Subclinical				80.0	47.2
Clinical				74.8	55.5

*AUC* Area under the Curve; *CI* AUC 95% confidence interval; *sensitivity* e.g., the specific data-driven ASD subscale was able to correctly classify 73.9% (subclinical cut-off score) or 68.6% (clinical cut-off score) of children with ASD as having an ASD classification; *specificity* e.g., the specific data-driven ASD subscale was able to correctly classify 46.9% (subclinical cut-off score) or 55.6% (clinical cut-off score) of children without ASD as having no ASD classification; *ASD profile* a combination of the Withdrawn/Depressed, Thought Problems, and Social Problems syndrome subscales; *WTP subscale* a combination of the Withdrawn/Depressed and Thought Problems syndrome subscales; *Specific ASD subscale* an ASD subscale consisting of separate CBCL 6-18 items; *NA* not available, due to there being no children with Conduct Disorder included in this sample

#### Comparison to the DSM-oriented subscales

Internal consistency of both the specific data-driven and clinician-expert ASD subscale was comparable to that of the DSM-oriented subscales in the second as well as in the third sample (Table 4). In both cross-validations samples, AUCs obtained for the DSM-oriented subscales were poor, with an exception for the Anxiety Problems subscale in Sample 2 (Tables 6 and 7). In general, the comparisons of different ROC curves revealed that both the specific data-driven and clinician-expert ASD subscale possessed similar levels of discriminative power as the DSM-oriented subscales (Tables 8 and 9; Appendix: Tables F and G). Considering performances in both the second and third sample, sensitivity seemed slightly higher and specificity seemed slightly lower regarding both the specific data-driven and clinician-expert ASD subscale when comparing to the DSM-oriented subscales (Tables 6 and 7).

**Table 8:** Textual description of AUC score comparisons between the specific data-driven ASD subscale and the other subscales

Comparison subscales	Sample 1	Sample 2	Sample 3
*Syndrome subscales*			
Withdrawn/depressed	<	<	<
Thought Problems	<	=	<
Social problems	<	=	=
*Previously developed ASD subscales*			
ASD profile by Biederman et al. (2010)	<	=	=
WTP subscale by Havdahl et al. (2016)	<	=	<
Specific ASD subscale by Ooi et al. (2011)	=	>	>
Specific ASD subscale by So et al. (2013)	<	=	=
*DSM-oriented subscales*			
Affective problems	=	=	=
Anxiety problems	=	=	=
Attention deficit/hyperactive problems	=	=	=
Oppositional defiant problems	>	=	=
Conduct problems	NA	<	NA
*Currently developed ASD subscales*			
Specific clinician-expert ASD subscale	<	=	=

For detailed information, see Appendix (Table F); < means that the specific data-driven ASD subscale performs better; = means that the specific data-driven ASD subscale performs equally; > means that the specific data-driven ASD subscale performs worse; NA = not available, due to there being no children with Conduct Disorder included in the concerning sample

**Table 9:** Textual description of AUC score comparisons between the specific clinician-expert ASD subscale and the other subscales

Comparison subscales	Sample 1	Sample 2	Sample 3
*Syndrome subscales*			
Withdrawn/depressed	<	<	<
Thought Problems	<	=	<
Social problems	<	=	=
*Previously developed ASD subscales*			
ASD profile by Biederman et al. (2010)	<	=	=
WTP subscale by Havdahl et al. (2016)	<	=	<
Specific ASD subscale by Ooi et al. (2011)	=	>	>
Specific ASD subscale by So et al. (2013)	=	=	=
*DSM-oriented subscales*			
Affective Problems	=	=	<
Anxiety Problems	=	=	=
Attention Deficit/Hyperactive Problems	>	=	=
Oppositional Defiant Problems	>	=	=
Conduct Problems	NA	<	NA

For detailed information, see Appendix (Table G); < means that the specific clinician-expert ASD subscale performs better; = means that the specific clinician-expert ASD subscale performs equally; > means that the clinician-expert ASD subscale performs worse; NA = not available, due to there being no children with Conduct Disorder included in the concerning sample

## Discussion

In this study, we used a data-driven and a clinician-expert approach to develop a subscale for the school-age CBCL to screen for ASD, consisting of separate items. Both the specific data-driven and clinician-expert ASD subscale—along with the syndrome subscales that in prior research have been associated with ASD, the ASD subscales that have been developed in previous studies, and the widely used DSM-oriented subscales—were validated as well as cross-validated in two truly independent samples.

Overall, our results demonstrated that the currently developed ASD subscales had a better ability to identify children with ASD compared to the Withdrawn/Depressed, Thought Problems, and the Social Problems syndrome subscales, as well as combinations of these syndrome subscales [i.e., the ASD profile developed by Biederman et al. (2010) and the WTP subscale developed by Havdahl et al. (2016)]. This result is in line with that of Deckers et al. (2020), who found that the specific ASD subscales developed by Ooi et al. (2011) and So et al. (2013) had a better capacity to differentiate between children with and without ASD compared to the (combinations of) syndrome subscales. This confirms the need for an ASD subscale based on individual school-age CBCL items, instead of relying on (combinations of) syndrome subscales. Although the currently developed ASD subscales had a higher internal consistency, they seemed to have a similar potential to discriminate between children with and without ASD as the specific ASD subscales of Ooi et al. (2011) and So et al. (2013), when considering the ROC analyses. This is not surprising, given the considerable item overlap between those and the currently developed ASD subscales. However, some of the statistical AUC comparisons indicated that out of all specific ASD subscales for the school-age CBCL, the data-driven had the highest discriminative power. Moreover, our results showed that the currently developed ASD subscales performed equivalently to the DSM-oriented subscales, with comparable Cronbach’s Alpha and AUC scores. Lastly, the currently developed ASD subscales showed high sensitivity, but relatively low specificity (particularly for the subclinical range). However, high sensitivity may be considered more important—especially during middle school and adolescence, when ASD symptoms might be subtler and more heterogeneous (Bal et al., 2019). Thus, our results suggest that the school-age CBCL seems as appropriate to screen for ASD as for other disorders (i.e., affective disorders, anxiety disorders, ADHD, ODD, and CD) and that when it comes to identifying children with ASD using this instrument, the specific data-driven subscale seems to be the best choice out of all examined ASD subscales for the school-age CBCL.

Thus, the currently developed ASD subscales for the school-age CBCL performed similarly to ASEBA’s DSM-oriented subscales. It should be noted, however, that compared to instruments that screen for ASD explicitly, their discriminative power was lower. For instance, when validating three ASD screeners—the Social Communication Questionnaire (SCQ; Berument et al., 1999), the Social Responsiveness Scale (SRS; Constantino & Gruber 2005), and the Children’s Communication Checklist (CCC; Bishop, 1998)—in a sample of children with IQ scores higher than 70, Charman et al. (2007) found levels of discriminative power that were somewhat higher compared to those we found for the currently developed ASD subscales in Sample 1, but clearly superior to those we found for the currently developed ASD subscales in the cross-validation samples (i.e., AUCs of at least 0.80 and sensitivity/specificity rates of at least 77%). A possible explanation for the relatively low sensitivity and/or specificity scores for the currently developed ASD subscales [as well as for the specific ASD subscales of Ooi et al. (2011) and So et al. (2013)], first of all, is that these are part of a broad and general screening questionnaire, instead of developed as a stand-alone questionnaire to specifically screen for ASD (related problems), such as the SRS, SCQ, and CCC. Second, the selected school-age CBCL items for the specific ASD subscales do not cover every aspect of ASD (e.g., there are no items included on hyper- or hypo-reactivity to sensory input) or might be too vague to describe ASD symptoms (e.g., repeats certain acts over and over, compulsions). Interestingly, the DSM-oriented Autism Spectrum Problems subscale for the preschool-age version of the CBCL (CBCL 1.5-5; Appendix: Table H) includes some items that are more ASD-specific (e.g., rocks head, body) and its psychometric properties have found to be very good. For instance, when discriminating between preschoolers with ASD and typically developing preschoolers, Muratori et al. (2011) found an AUC of 0.95, a sensitivity of 85%, and a specificity of 90%. However, when comparing to preschoolers with other psychiatric disorders, results (i.e., AUC = 0.81; sensitivity = 85%; specificity = 60%) were more similar to those we found for the currently developed ASD subscales in Sample 1. Third, the presentation of ASD symptoms might change over time. That is, symptoms may differ for children during early childhood, middle childhood, and adolescence (Bal et al., 2019). Perhaps, the items of the school-age CBCL do not reflect ASD symptom expression during middle childhood and adolescence as well as the items of the preschool-age CBCL do during early childhood. On the other hand, one could argue that because the Autism Spectrum Problems subscale (CBCL 1.5-5) is somewhat more ASD specific—with a few items being very typical for ASD or describing rather severe problem behavior—preschoolers with ASD that display subtler symptomatology and/or require less (parental) support might be missed. In the current study, we tried to account for changes in ASD symptom expression over time by—like ASEBA—considering different norms for children and adolescents. However, future (preferably longitudinal) studies are needed to explore the discriminative ability of the different specific ASD subscales for the CBCL across childhood (i.e., from the preschool to the adolescent years) and/or the lifespan—if one would be able to establish an ASD subscale for the Adult Self Report (ASR; Achenbach & Rescorla, 2003) as well. Lastly, another factor that might explain the relatively low specificity scores for the currently developed ASD subscales in particular, is that the majority of children in the first sample had an ADHD classification [as was the case in the study of Deckers et al. (2020)]. To wit, there is high symptom overlap and comorbidity between ASD and ADHD (e.g., 59%; Stevens et al., 2016). On the other hand, ASD might share symptom overlap and frequently co-exists with other disorders as well (i.e., the comorbidity rate between ASD and anxiety disorders is 40%; van Steensel et al., 2011). Therefore, we recommend more direct comparisons between children with ASD and children with other classifications, in order to evaluate the discriminative ability of the currently developed ASD subscales. Particularly, future studies should include larger numbers of children with internalizing classifications in clinical control groups, as these were less well represented in our samples.

Important to note is that, even though the specific ASD subscales for the school-age CBCL seem to have less discriminative power compared to instruments developed to explicitly screen for ASD (e.g., the SRS, SCQ, and CCC), both types of instruments might serve different purposes. To wit, such ASD screeners are rarely implemented in school settings due to their administration being quite time-consuming and expensive (So et al., 2013). Also, these disorder-specific instruments are hardly used to screen for ASD at intake in general mental health care centers (to which children are often [first] referred when they display social, emotional, and/or behavioral problems) due to intake sessions leaving little room for the administration of extra assessments focusing on merely one type of psychopathology (Deckers et al., 2020). However, community-based pediatric services, which often use the school-age CBCL to routinely screen for a broad range of (mental) health problems in children at specified moments during their development, could profit from an ASD subscale because it can easily be incorporated in the analysis of results. Also, in clinical settings, an ASD subscale can effortlessly be added to the analysis of the screening results, if the administration of the school-age CBCL is part of the standard procedure. Thus, the ASD subscale for the school-age CBCL can be used as a first exploration into possible ASD symptomology and when children score above the corresponding cut-off, instruments to explicitly screen for ASD can be administered to zoom in further. This way, the use of the school-age CBCL ASD subscale might save time, and therefore expenses. In future research, it would be interesting to compare the performance of the currently developed ASD subscales to that of explicit ASD screeners, to determine whether such disorder-specific screening instruments add significant value over and above a first screening with the school-age CBCL.

It was remarkable that in terms of item composition, although there was some overlap, the data-driven (i.e., based on parent reports) and clinician-expert (i.e., based on opinions of clinicians) approaches yielded different ASD subscales. Discrepancies between parent and clinician observations in the assessment of ASD symptoms, however, are not uncommon (e.g., de Bildt et al., 2004; Lemler, 2012; Neuhaus et al., 2018). An advantage of the data-driven approach might be that parents spend the most time with their children and are the first ones to detect certain problems, thus are the main informants with reference to their children’s (problem) behavior (Lemler, 2012). Yet, parent’s observations might be prone to over- or under-estimation, as they do not experience their children’s behavior in the school or clinical setting (Lemler, 2012), and may be biased due to perceived parenting stress (Schwartzman et al., 2021). A strength of the clinician-expert approach might be that clinicians are more trained in observing the heterogeneous nature of the ASD symptomatology, and have seen or worked with multiple children with ASD. To illustrate, Lord et al. (2006), who used both a parent- and a clinician-based diagnostic instrument to examine the stability of ASD diagnoses at ages two and nine, found that clinicians had a higher percentage of agreement in accurate diagnosis compared to parents. However, clinicians mainly observe children in the clinical setting, which might lead to their view on the functioning of children with ASD being somewhat one-sided (Lemler, 2012). Interestingly, Neuhaus et al. (2018) found that disagreement between parents and clinicians was bigger when, amongst others, children had higher IQ-scores and displayed more adaptive behavior. Thus, considering the characteristics of the children included in Sample 1, following both a data-driven and a clinician-expert approach—hence constructing two different specific ASD subscales for the school-age CBCL—was the most thorough way of conducting the current study. It should be noted that our Clinicians Sample only included 15 participants, who were all employed at the same academic mental health care center. A larger-scale, international replication of the clinician-expert approach—like the one used by ASEBA when developing the DSM-oriented subscales—is needed to determine whether this or the data-driven approach is most valid for constructing a specific ASD subscale for the school-age CBCL.

A somewhat disappointing finding was that the discriminative power of not only the specific data-driven and clinician-expert ASD subscales, but also that of all other investigated syndrome subscales, previously developed ASD subscales, and ASEBA’s DSM-oriented subscales (to screen for depression, anxiety, ADHD, ODD, and CD), was lower in the cross-validation samples than in the first sample and samples used in previous validation research. For instance, Deckers et al. (2020) found that the ability to identify children with ASD of the (combinations of) syndrome subscales ranged from poor to fair, and that this ability of the specific ASD subscales of Ooi et al. (2011) and So et al. (2013) ranged from fair to good. Also, Ebestuani et al. (2010) found that the discriminative power of the different DSM-oriented subscales ranged from fair to good. The relatively low AUC, sensitivity, and specificity values in our cross-validation samples might be due to several methodological factors, such as different sample characteristics (e.g., level of intelligence, social economic status, and family situation) or different diagnostic and screening procedures applied within the participating mental health care centers. Although a range of methods (i.e., interviews, observations, questionnaires, and/or psychiatric/neuropsychological evaluations) was available to be used during the diagnostic process, there was no standardized protocol for establishing DSM classifications, thus which methods were applied could vary per mental health care center and/or per child. In addition, a standardized protocol to screen for comorbidity was lacking. This could have influenced both classification and comorbidity rates.

Strengths of the current study include the use of both a data-driven and a clinician-expert approach in constructing specific ASD subscales for the school-age CBCL, the large number of participants, the comparisons to ASEBA’s DSM-oriented subscales, and the use of two truly independent cross-validation samples. That is, we explicitly chose to use truly independent cross-validation samples over applying random subsampling (i.e., combining all data and then splitting it in half), as we wanted to explore how well a specific ASD subscale constructed within one treatment center would perform in others. In contrast, random subsampling would have led to the development/validation and cross-validation samples being rather similar, which—to our opinion—would not properly test generalizability to other treatment centers (with other sample characteristics). Although applying a resampling approach might have ensured more robustness of our specific ASD subscales in cross-validation efforts, we chose to retain the samples for development/validation and cross-validation as distinct groups, as the ultimate goal was to construct an appropriate specific ASD subscale that can be used in different clinical and pediatric settings. As such, we have chosen the sample of one treatment center as the development/validation sample (i.e., Sample 1, as this one included the most children with ASD and contained the most descriptive and clinical information), and used the other samples as truly independent cross-validation samples.

Some shortcomings—aside from the majority of children in Sample 1 having an ADHD classification, the relatively small size of the Clinicians Sample, and the varying screening and diagnostic procedures applied within the participating mental health care centers—need to be acknowledged as well. First, as Sample 1 included children that had been referred to the mental health care center between 2009 and 2020, some of them had a DSM-IV-TR instead of a DSM-5 ASD classification. However, Kulage et al. (2020), who conducted a 5-year follow-up systematic review and meta-analysis in which they included 33 studies, found that 79% of children with a DSM-IV-TR classification still met the DSM-5 criteria for ASD. Also, in the DSM-5, the American Psychiatric Association states that individuals with a well-established DSM-IV-TR classification of autistic disorder, Asperger’s disorder, or pervasive developmental disorder not otherwise specified, should be given the DSM-5 classification of ASD (APA, 2013). Therefore, we decided not to exclude the children with a DSM-IV-TR ASD classification. Second, the perspectives of teachers were not considered in the development and validation of our specific ASD subscales. This could have been of great value, as teachers are provided with lots of opportunities to observe children in social situations, especially during interactions with their peers. It should be noted, however, that Deckers et al. (2020) found that parents were better informants when identifying children with ASD compared to teachers. The authors argued that this may be explained by parents being able to observe their child in various settings and over time. Besides, through communication with the teacher, they might also be able to assess how their child is behaving at school.

A remaining question for future research is whether the currently and previously developed specific ASD subscales are culture-bound. To wit, previous research has found cultural differences regarding ASD symptom expression (e.g., Matson et al., 2011) and although the school-age CBCL has been validated for use in many cultures (e.g., Rescorla et al., 2007), the specific ASD subscales have mostly been developed and/or validated using samples predominantly consisting of Western participants.

In conclusion, the results of this study indicated that out of all developed ASD subscales for the school-age CBCL, the specific data-driven seems to have the best potential to screen for ASD during middle childhood and adolescence. Also, this subscale has a similar screening potential as the DSM-oriented subscales developed by ASEBA, which have been widely used for nearly two decades. It should be noted that all examined subscales (including ASEBA’s DSM-oriented subscales) showed rather poor discriminative power in the cross-validation samples. However, considering the possible benefits for both pediatric and clinical practice, we encourage our colleagues to continue the validation of this specific ASD subscale for the school-age CBCL.

## Supplementary Information

Below is the link to the electronic supplementary material.

Supplementary material 1 (DOCX 51.1 kb)
