# Supplementary material for: The Development and Validation of a Subscale for the School-Age Child Behavior CheckList to Screen for Autism Spectrum Disorder
Source: J Autism Dev Disord. 2022 Feb 15;53(3):1034–52. doi: 10.1007/s10803-022-05465-7 (PMC9986212; doi:10.1007/s10803-022-05465-7)
Supplement: Supplementary file 1 — Supplementary material 1 (DOCX 51.1 kb) [file 10803_2022_5465_MOESM1_ESM.docx]

**Appendix**

**Table A.** *Cut-off scores for the Specific ASD Subscales Developed in the Current Study*

|  | Specific data-driven ASD subscale | Specific clinician-expert ASD subscale |
| --- | --- | --- |
| Max score | 30 | 46 |
| *Subclinical cut-offs* |  |  |
| Girls |  |  |
| 12- | 6 | 12 |
| 12+ | 8 | 11 |
| Boys |  |  |
| 12- | 7 | 11 |
| 12+ | 7 | 11 |
| *Clinical cut-offs* |  |  |
| Girls |  |  |
| 12- | 8 | 13 |
| 12+ | 10 | 13 |
| Boys |  |  |
| 12- | 8 | 12 |
| 12+ | 8 | 12 |

**Table B.** *Cut-off Scores for the CBCL 6-18 Syndrome Subscales (Achenbach & Rescorla, 2001)*

|  | Withdrawn/depressed | Thought Problems | Social Problems |
| --- | --- | --- | --- |
| Max score | 16 | 30 | 22 |
| *Subclinical cut-offs* |  |  |  |
| Girls |  |  |  |
| 12- | 5 | 5 | 8 |
| 12+ | 6 | 5 | 6 |
| Boys |  |  |  |
| 12- | 4 | 6 | 7 |
| 12+ | 6 | 5 | 6 |
| *Clinical cut-offs* |  |  |  |
| Girls |  |  |  |
| 12- | 7 | 7 | 9 |
| 12+ | 8 | 7 | 9 |
| Boys |  |  |  |
| 12- | 6 | 7 | 10 |
| 12+ | 8 | 8 | 9 |

**Table C.** *Cut-off scores in Various Studies for the Previously Developed ASD Subscales*

|  | Study | Max score | Cut-off score |
| --- | --- | --- | --- |
| ASD profile by Biederman et al. (2010) | Biederman et al. (2010) | 300* | 195* |
| WTP subscale by Havdahl et al. (2016) | Havdahl et al. (2016) | 200* | 124* |
| Specific ASD subscale of Ooi et al. (2011) | Deckers et al. (2020) | 18** | 4** |
| Specific ASD subscale of So et al. (2013) | Deckers et al. (2020) | 20** | 5** |

*Note.* * = T-score; ** = Raw score; ASD profile = a combination of the Withdrawn/Depressed, Thought Problems, and Social problems syndrome subscales; WTP subscale = a combination of the Withdrawn/Depressed and Thought Problems syndrome subscales; Specific ASD subscale = an ASD subscale consisting of separate CBCL 6-18 items

**Table D.** *Cut-off Scores for the DSM-oriented Subscales (Achenbach et al., 2001)*

|  | Affective Problems | Anxiety Problems | Attention/Hyperactive Problems | Oppositional Defiant Problems | Conduct Problems |
| --- | --- | --- | --- | --- | --- |
| Max score | 26 | 12 | 14 | 10 | 34 |
| *Subclinical cut-offs* |  |  |  |  |  |
| Girls |  |  |  |  |  |
| 12- | 5 | 5 | 8 | 6 | 5 |
| 12+ | 6 | 5 | 7 | 6 | 6 |
| Boys |  |  |  |  |  |
| 12- | 5 | 4 | 9 | 6 | 6 |
| 12+ | 6 | 4 | 8 | 6 | 7 |
| *Clinical cut-offs* |  |  |  |  |  |
| Girls |  |  |  |  |  |
| 12- | 7 | 6 | 10 | 7 | 7 |
| 12+ | 9 | 6 | 9 | 8 | 11 |
| Boys |  |  |  |  |  |
| 12- | 7 | 6 | 11 | 7 | 9 |
| 12+ | 8 | 5 | 11 | 8 | 13 |

**Table E.** *Comparison of the Specific Data-Driven and Clinician-Expert ASD subscales Regarding the Number of Children Classified as ‘Having ASD’ or ‘Not Having ASD’*

|  | **Sub-clinical cut-off score** | | | | | |  | **Clinical cut-off score** | | | | |
| --- | --- | --- | --- | --- | --- | --- | --- | --- | --- | --- | --- | --- |
| **S** | Total *N* | *N* = ASD by both subscales (%) | *N* = ASD, by one of the subscales (%) | *N* = ASD, by the data-driven only (%) | *N* = ASD, by the clinician-expert only (%) | *N* = no ASD, by both subscales (%) |  | *N* = ASD by both subscales (%) | *N* = ASD, by one of the subscales (%) | *N* = ASD, by the data-driven only (%) | *N* = ASD, by the clinician-expert only (%) | *N* = no ASD, by both subscales (%) |
| **1** | 1662 | 706 (42.5%) | 252 (15.2%) | 153 (9.2%) | 99 (6.0%) | 704 (42.4%) |  | 552 (33.2%) | 227 (13.7%) | 96 (5.8%) | 131 (7.9%) | 883 (53.1%) |
| **2** | 1893 | 989 (52.2%) | 248 (13.1%) | 64 (3.4%) | 182 (9.6%) | 656 (34.7%) |  | 781 (41.3%) | 284 (15.0%) | 44 (2.3%) | 240 (12.7%) | 828 (43.7%) |
| **3** | 583 | 297 (50.9%) | 82 (14.1%) | 27 (4.6%) | 55 (9.4%) | 204 (35.0%) |  | 251 (43.1%) | 84 (14.4%) | 40 (6.9%) | 48 (8.2%) | 248 (42.5%) |

*Note.* % = valid percent; S = sample; Total *N* = number of children that were classified by both the specific data-driven and clinician-expert ASD subscale

**Table F.** *AUC Score Comparisons between the Specific Data-Driven ASD Subscale and the Other Subscales*

|  | **Sample 1 (*N* = 1666)** | | | | |  | **Sample 2 (*N* = 2445)** | | | | |  | **Sample 3 (*N* = 886)** | | | | |
| --- | --- | --- | --- | --- | --- | --- | --- | --- | --- | --- | --- | --- | --- | --- | --- | --- | --- |
|  | *AUC difference(CI)* | *SE* | *Z* | *p* | *d* |  | *AUC difference(CI)* | *SE* | *Z* | *p* | *d* |  | *AUC difference(CI)* | *SE* | *Z* | *p* | *d* |
| **Syndrome subscales** |  |  |  |  |  |  |  |  |  |  |  |  |  |  |  |  |  |
| Withdrawn/depressed^1^ | .06(.03 – .08) | .01 | 4.67 | <.001 | .18 |  | .09(.05 – .12) | .02 | 4.65 | <.001 | .19 |  | .08(.04 – .11) | .02 | 4.10 | <.001 | .15 |
| Thought Problems^1^ | .14(.11 – .18) | .02 | 8.49 | <.001 | .40 |  | .02(-.02 –.06) | .02 | -1.18 | .239 | .08 |  | .06(.02 – .10) | .02 | 2.94 | .003 | .15 |
| Social Problems^1^ | .09(.06 – .12) | .01 | 6.30 | <.001 | .27 |  | .01(-.03 – .05) | .02 | .57 | .569 | .04 |  | .01(-.02 – .04) | .02 | .53 | .598 | .01 |
|  |  |  |  |  |  |  |  |  |  |  |  |  |  |  |  |  |  |
| **Previously developed ASD subscales** |  |  |  |  |  |  |  |  |  |  |  |  |  |  |  |  |  |
| ASD profile by Biederman et al. (2010)^1^ | .04(.02 – .05) | .01 | 4.36 | <.001 | .11 |  | .00(-.03 – .03) | .01 | .06 | .949 | .01 |  | .01(-.02 – .03) | .01 | .74 | .462 | .02 |
| WTP subscale by Havdahl et al. (2016)^1^ | .06(.03 – .08) | .01 | 5.25 | <.001 | .17 |  | .01(-.02 – .05) | .02 | .82 | .415 | .02 |  | .05(.02 – .08) | .02 | 3.24 | .001 | .12 |
| Specific ASD subscale by Ooi et al. (2011)^1^ | .01(-.01 – .02) | .01 | .78 | .438 | .02 |  | .05(.02 – .07) | .02 | 2.95 | .003 | .09 |  | .04(.01 – .07) | .01 | 2.95 | .003 | .10 |
| Specific ASD subscale by So et al. (2013)^1^ | .02(.01 – .04) | .01 | 3.37 | <.001 | .07 |  | .00(-.02 – .03) | .01 | .43 | .671 | .00 |  | .02(-.00 – .03) | .01 | 1.75 | .080 | .04 |
|  |  |  |  |  |  |  |  |  |  |  |  |  |  |  |  |  |  |
| **DSM-oriented subscales** |  |  |  |  |  |  |  |  |  |  |  |  |  |  |  |  |  |
| Affective Problems^2^ | .03(-.00 –.06) | .03 | 1.05 | .294 | .10 |  | .05(-.01 – .09) | .04 | 1.08 | .279 | .12 |  | .06(.00 – .12) | .04 | 1.44 | .151 | .15 |
| Anxiety Problems^2^ | .03(-.00 – .06) | .02 | 1.36 | .173 | .09 |  | .04(-.04 – .12) | .04 | 1.93 | .054 | .24 |  | .02(.01 – .03) | .03 | .29 | .769 | .02 |
| Attention Deficit/Hyperactive Problems^2^ | .02(-.01 – .05) | .02 | 1.32 | .186 | .08 |  | .06(.00 – .12) | .03 | 1.86 | .062 | .13 |  | .00(-.00 – .00) | .03 | .07 | .941 | .01 |
| Oppositional Defiant Problems^2^ | .06( -.01 – .13) | .03 | 2.33 | .020 | .23 |  | .04(-.00 – .08) | .03 | 1.47 | .143 | .10 |  | .02(.01 – .03) | .02 | .53 | .596 | .05 |
| Conduct Problems^2^ | NA | NA | NA | NA | NA |  | .08(-.78 – .90) | .04 | 2.04 | .042 | .19 |  | NA | NA | NA | NA | NA |
|  |  |  |  |  |  |  |  |  |  |  |  |  |  |  |  |  |  |
| **Currently developed ASD subscales** |  |  |  |  |  |  |  |  |  |  |  |  |  |  |  |  |  |
| Specific clinician-expert ASD subscale^1^ | .02(.00 – .03) | .01 | 2.21 | .027 | .06 |  | .00(-.03 –.03) | .01 | .02 | .981 | .00 |  | .00(-.02 – .03) | .01 | .27 | .784 | .09 |

*Note.* ^1^ DeLong et al.’s test (1988) was used; ^2^ a comparison of different ROC curves was conducted; ASD profile = a combination of the Withdrawn/Depressed, Thought Problems, and Social Problems syndrome subscales; WTP subscale = a combination of the Withdrawn/Depressed and Thought Problems syndrome subscales; Specific ASD subscale = an ASD subscale consisting of separate CBCL 6-18 items; NA = not available, due to there being no children with Conduct Disorder included in the concerning sample

**Table G.** *AUC Score Comparisons between the Specific Clinician-Expert ASD Subscale and the Other Subscales*

|  | **Sample 1 (*N* = 1666)** | | | | |  | **Sample 2 (*N* = 2445)** | | | | |  | **Sample 3 (*N* = 886)** | | | | |
| --- | --- | --- | --- | --- | --- | --- | --- | --- | --- | --- | --- | --- | --- | --- | --- | --- | --- |
|  | *AUC difference(CI)* | *SE* | *Z* | *p* | *d* |  | *AUC difference(CI)* | *SE* | *Z* | *p* | *d* |  | *AUC difference(CI)* | *SE* | *Z* | *p* | *d* |
| **Syndrome subscales** |  |  |  |  |  |  |  |  |  |  |  |  |  |  |  |  |  |
| Withdrawn/depressed^1^ | .04(.02 – .07) | .01 | 3.23 | .001 | .13 |  | .08(.04 – .12) | .02 | 3.66 | <.001 | .20 |  | .08(.04 – .12) | .02 | 4.06 | <.001 | .25 |
| Thought Problems^1^ | .13(.10 – .16) | .02 | 8.06 | <.001 | .35 |  | .02(-.02 – .06) | .02 | .88 | .378 | .08 |  | .06(.02 – .11) | .02 | 2.80 | .005 | .24 |
| Social Problems^1^ | .08(.05 – .10) | .01 | 5.52 | .001 | .22 |  | .00(-.04 – .04) | .02 | .10 | .923 | .04 |  | .00(-.03 – .04) | .02 | .20 | .843 | .09 |
|  |  |  |  |  |  |  |  |  |  |  |  |  |  |  |  |  |  |
| **Previously developed ASD subscales** |  |  |  |  |  |  |  |  |  |  |  |  |  |  |  |  |  |
| ASD profile by Biederman et al. (2010)^1^ | .02(.01 – .03) | .01 | 2.77 | .006 | .06 |  | .01(-.02 –.04) | .01 | .65 | .516 | .01 |  | .01(-.01 – .03) | .01 | .72 | .470 | .12 |
| WTP subscale by Havdahl et al. (2016)^1^ | .04(.02 – .06) | .01 | 3.85 | <.001 | .11 |  | .01(-.02 –.05) | .02 | .67 | .504 | .02 |  | .05(.02 – .08) | .01 | 3.21 | .001 | .21 |
| Specific ASD subscale by Ooi et al. (2011)^1^ | .01(-.01 – .03) | .01 | 1.12 | .263 | .03 |  | .05(.02 – .09) | .02 | 3.11 | .002 | .09 |  | .04(.01 – .07) | .02 | 2.39 | .017 | .01 |
| Specific ASD subscale by So et al. (2013)^1^ | .00(-.02 – .03) | .01 | .45 | .655 | .01 |  | .01(-.03 – .04) | .02 | .31 | .756 | .00 |  | .02(-.02 –.05) | .02 | .98 | .325 | .05 |
|  |  |  |  |  |  |  |  |  |  |  |  |  |  |  |  |  |  |
| **DSM-oriented subscales** |  |  |  |  |  |  |  |  |  |  |  |  |  |  |  |  |  |
| Affective Problems^2^ | .04(-.01 – .09) | .03 | 1.74 | .083 | .15 |  | .05(.05 – .06) | .05 | .10 | .318 | .11 |  | .10(.01 – .19) | .04 | 2.22 | .026 | .24 |
| Anxiety Problems^2^ | .01(.00 – .02) | .02 | .47 | .638 | .03 |  | .09(-.00 – 18) | .05 | 1.83 | .068 | .23 |  | .03(-.00 – .06) | .04 | .77 | .444 | .07 |
| Attention Deficit/Hyperactive Problems^2^ | .04(-.01 – .09) | .02 | 2.31 | .021 | .14 |  | .05(.00 – .10) | .03 | 1.65 | .100 | .13 |  | .03(-.00 – .06) | .03 | 1.09 | .274 | .09 |
| Oppositional Defiant Problems^2^ | .08(0.01 – .17) | .03 | 3.00 | .003 | .29 |  | .04(-.00 – .08) | .03 | 1.40 | .161 | .10 |  | .02(.00 – .04) | .04 | .40 | .691 | .04 |
| Conduct Problems^2^ | NA | NA | NA | NA | NA |  | .09(.01 – .17) | .04 | 1.99 | .046 | .19 |  | NA | NA | NA | NA | NA |

*Note.* ^1^ DeLong et al.’s test (1988) was used; ^2^ a comparison of different ROC curves was conducted; ASD profile = a combination of the Withdrawn/Depressed, Thought Problems, and Social Problems syndrome subscales; WTP subscale = a combination of the Withdrawn/Depressed and Thought Problems syndrome subscales; Specific ASD subscale = an ASD subscale consisting of separate CBCL 6-18 items; NA = not available, due to there being no children with Conduct Disorder included in the concerning sample

**Table H.** *Items of the Specific Data-Driven ASD Subscale (CBCL 6-18), the Specific Clinician-Expert ASD Subscale (CBCL 6-18), and Autism Spectrum Problems Subscale (CBCL 1.5-5)*

| **Specific data-driven ASD subscale (*n* = 15)** | **Specific clinician-expert ASD subscale (*n* = 23)** | **Autism Spectrum Problems subscale (*n* = 13)** |
| --- | --- | --- |
| Acts too young for his/her age | Acts too young for his/her  age | Afraid to try new things |
| There is little that he/she enjoys | There is little that he/she enjoys | Avoids eye contact |
| Cannot get his/her mind off  certain thoughts; obsessions | Bowel movements outside toilet | Can’t stand things out of place |
| Clings to adults or too dependent | Cannot get his/her mind off  certain thoughts; obsessions | Disturbed by change |
| Daydreams or gets lost in  his/her thoughts | Daydreams or gets lost in  his/her thoughts | Doesn’t answer |
| Doesn’t get along with other kids | Doesn’t eat well | Doesn’t get along with peers |
| Would rather be alone than with  others | Doesn’t get along with other  kids | Rocks head, body |
| Poorly coordinated or clumsy | Fears certain animals, situations, or places other than school | Unresponsive to affection |
| Prefers being with younger kids | Gets teased a lot | Little affection |
| Repeats certain acts over and  over; compulsions | Would rather be alone than with  others | Speech problem |
| Too shy or timid | Not liked by other kids | Strange behavior |
| Stares blankly | Prefers being with younger kids | Upset by new |
| Strange behavior | Repeats certain acts over and  over; compulsions | Withdrawn |
| Underactive, slow moving, or  lacks energy | Secretive, keeps things to self |  |
| Withdrawn, doesn’t get involved  with others | Too shy or timid |  |
|  | Stares blankly |  |
|  | Stores up too many things he/she  does not need |  |
|  | Strange behavior |  |
|  | Strange ideas |  |
|  | Stubborn, sullen, or irritable |  |
|  | Sudden changes in mood or feelings |  |
|  | Temper tantrums or hot temper |  |
|  | Withdrawn, doesn’t get involved  with others |  |
